# Ibandronic Acid Induced Orbital Inflammation and Concurrent Anterior Uveitis - A Rare Presentation

**DOI:** 10.22336/rjo.2025.42

**Published:** 2025

**Authors:** Jayasri Periyandavan, Amudha Purushothaman, Vijhayapriya Thanasekaran, Ezhilvathani T Namaskaram

**Affiliations:** Indira Gandhi Medical College & Research Institute, Puducherry, India

**Keywords:** Ibandronic acid, bisphosphonates, orbital inflammation, anterior uveitis, intravenous methyl prednisolone, ALC = Anterior lens capsule, CT = Computed tomography, TNF = Tumour necrosis factor

## Abstract

**Aim:**

To report the first case of orbital inflammation with concurrent anterior uveitis induced by oral Ibandronate, a bisphosphonate used for osteoporosis and other bone disorders.

**Material and methods:**

A 55-year-old female, with a history of diabetes and hypertension, developed bilateral eye pain, redness, and photophobia two days after taking oral Ibandronate for a thoracic spine fracture. Examination revealed reduced visual acuity, anterior uveitis at presentation, and bilateral proptosis two days later. The imaging revealed diffuse bilateral orbital inflammation.

**Results:**

After discontinuing the drug, the patient was treated with intravenous methylprednisolone, leading to significant improvement in ocular symptoms.

**Discussion:**

Bisphosphonates, including Ibandronate, can cause ocular adverse effects such as uveitis and orbital inflammation. The pathophysiology is linked to immune modulation via gamma T-cell activation. Most cases respond to discontinuation of drug and steroid administration. The risk-benefit ratio of steroid administration has to be weighed, as it can worsen osteoporosis. Early detection and timely discontinuation of the drug can avoid sight-threatening complications.

**Conclusion:**

This case underscores the importance of early recognition and prompt management of ocular complications, particularly as the use of bisphosphonates continues to increase.

## Introduction

Bisphosphonates are being increasingly used nowadays for the treatment of post-menopausal osteoporosis, Paget’s disease, bony metastasis, and osteolytic bony disorders. The commonly used bisphosphonates include zoledronic acid (also known as zoledronate), risedronate, pamidronate, ibandronate, and alendronate. They inhibit osteoclastic bone resorption.

**Fig. 1A:**
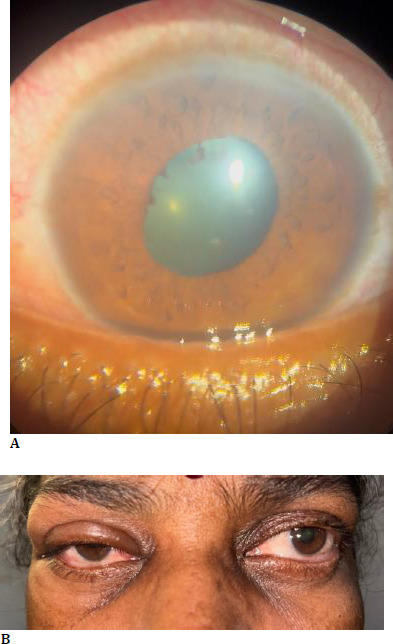
Clinical photograph showing features of anterior uveitis; **B**. Clinical photograph showing bilateral proptosis with conjunctival chemosis

We report the first case of Ibandronic acid-induced orbital inflammation with concurrent anterior uveitis. The patient was managed promptly with intravenous steroids after discontinuation of ibandronate. This is the first reported case of orbital inflammation with anterior uveitis (**Fig. 1A**) following the administration of oral ibandronate. Since bisphosphonates are being used for various conditions, it is the prerogative of all physicians to identify the ocular side effects and prevent sight-threatening complications.

## Case report

**Fig. 2A:**
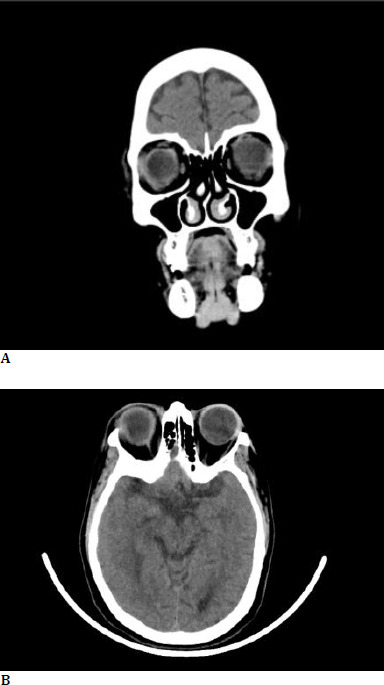
Computed tomography image - coronal section showing an inflamed lacrimal gland and bulky extraocular muscles; **B**. Computed tomography image - axial section showing an inflamed lacrimal gland

**Fig. 3:**
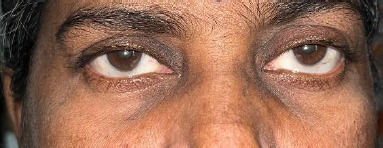
Clinical photograph showing complete resolution of proptosis and conjunctival chemosis

A 55-year-old female, known diabetic and hypertensive, presented with the sudden onset of pain, redness, and photophobia in both eyes for 2 days. She had a history of intake of oral Ibandronic acid for the fracture of the thoracic spine secondary to osteoporosis. Two days later, she developed the complaints mentioned above and discontinued the drug. On presentation, her visual acuity was OD 3/60 and OS 5/60, with no further improvement. Both eyes had lid edema, conjunctival congestion, a few pigments on the endothelium, 2+ cells in the anterior chamber, posterior synechiae in 2-3 non-contiguous clock hours, and pigments on the ALC with early lens changes. Posterior segment examination revealed BE disc pallor, peripapillary atrophy with myopic macular degeneration. Axial length was 26 mm and 27 mm in both eyes, respectively. Intraocular pressure was 26 mmHg in RE and 13 mmHg in LE. She was admitted and started on topical steroids, antibiotics, cycloplegics, and one anti-glaucoma medication, and worked up for immunological and infectious causes of uveitis, which turned negative. Two days later, she developed bilateral proptosis, increased lid edema, conjunctival chemosis, and restricted ocular motility. Urgent neuroimaging (CT scan of the brain and orbit) was performed, which revealed bilateral proptosis (**Fig. 1B**) with prominent medial and inferior rectus muscles, intraorbital fat stranding, and a relatively prominent right lacrimal gland, suggestive of orbital inflammation (**Fig. 2A, B**). She was started on intravenous methylprednisolone on the same day. The patient’s proptosis and ocular motility drastically improved (**Fig. 3**). Following three days of IV methylprednisolone, she was started on oral prednisolone.

## Results

The patient showed significant improvement of her ocular symptoms and signs after prompt discontinuation of the drug and initiation of intravenous methylprednisolone. The clear chronological association and improvement upon discontinuation of the offending drug suggest an adverse drug reaction.

## Discussion

Bisphosphonates are being increasingly used nowadays for the treatment of post-menopausal osteoporosis, Paget’s disease, bony metastasis, and osteolytic bony disorders [1]. The commonly used bisphosphonates include zoledronic acid (also known as zoledronate), risedronate, pamidronate, ibandronate, and alendronate. They inhibit osteoclastic bone resorption. Ocular adverse effects, including conjunctivitis, scleritis, keratitis, anterior uveitis, orbital inflammation, and optic neuritis, have been reported [2]. The proposed mechanism of causing ocular inflammation involves activating Gamma T cells by inhibiting farnesyl pyrophosphate synthase, which leads to the accumulation of isopentenyl diphosphate and dimethylallyl diphosphate, resulting in the release of proinflammatory cytokines such as IL-6 and TNF-alpha [3-6].

Bartalay et al. [7] recently published a review of 43 cases of orbital inflammation associated with bisphosphonates, with 67% of the cases occurring following the intravenous infusion of zoledronic acid. The majority of cases were unilateral, and 9 cases were reported to have concurrent uveitis. One case of anterior ischemic optic neuropathy with orbital inflammation has been reported following zoledronate infusion for metastatic prostate cancer [8]. Orbital imaging of previous case reports revealed diffuse orbital inflammation with fat stranding, indicating more extensive involvement than localized conditions such as myositis or dacryoadenitis. The mean duration of symptom onset following drug administration was within 72 hours in most cases. Prompt discontinuation of the offending drug, accompanied by corticosteroid administration, resulted in improvement in all cases.

We report this case of bilateral acute anterior uveitis with orbital inflammation following oral ibandronate. The clear chronological correlation between the use of ibandronate and the onset of uveitis and orbital inflammation suggests that it is drug-induced. The drastic response to discontinuation of drug and steroid infusion suggests it is drug-induced. We also analysed the adverse drug reaction probability using the Naranjo score [9], which was 8, indicating a positive correlation.

The use of corticosteroids is a double-edged sword that can worsen osteoporosis. The dose should be balanced against the risks and benefits. Yang et al. [10] reinstalled the treatment with a lower potency bisphosphonate (pamidronate instead of zoledronate) alongside systemic steroids, and reduced adverse symptoms were observed with each subsequent treatment, suggesting a tolerance effect. Hence, bisphosphonate use is not entirely contraindicated and can be reconsidered in light of the necessity of the therapy.

## Conclusion

Early recognition of ocular adverse events and prompt discontinuation of the drug with steroid treatment can prevent sight-threatening complications. Given the increasing use of bisphosphonates for various conditions, ranging from osteoporosis to metastatic osteolytic cancers, there is likely to be an increase in the incidence of ocular adverse events in the future. This warrants sensitization of the treating fraternity regarding ocular complications for early recognition and treatment.
